# Mechanical Stress-Induced IGF-1 Facilitates col-I and col-III Synthesis via the IGF-1R/AKT/mTORC1 Signaling Pathway

**DOI:** 10.1155/2021/5553676

**Published:** 2021-12-06

**Authors:** Bin Yan, Canjun Zeng, Yuhui Chen, Minjun Huang, Na Yao, Jie Zhang, Bo Yan, Jiajun Tang, Liang Wang, Zhongmin Zhang

**Affiliations:** ^1^Department of Spine Surgery, Center for Orthopedic Surgery, The Third Affiliated Hospital of Southern Medical University, Guangzhou, Guangdong, China; ^2^Department of Foot and Ankle Surgery, Center for Orthopedic Surgery, The Third Affiliated Hospital of Southern Medical University, Guangzhou, Guangdong, China; ^3^Department of Traumatic Surgery, Center for Orthopedic Surgery, The Third Affiliated Hospital of Southern Medical University, Guangzhou, Guangdong, China; ^4^Guangdong Food and Drug Vocational-Technical School, Guangzhou, Guangdong, China; ^5^Department of Spine Surgery, Nanfang Hospital of Southern Medical University, Guangzhou, Guangdong, China

## Abstract

Mechanical stress promotes human ligamentum flavum cells (LFCs) to synthesize multitype collagens, leading to ligamentum flavum hypertrophy (LFH). However, the mechanism of mechanical stress in the formation of collagen remains unclear. Therefore, we investigated the relationship between mechanical stress and collagen synthesis in the present study. First, LFCs were isolated from 9 patients and cultured with or without mechanical stress exposure for different times. IGF-1, collagen I (col-I), and collagen III (col-III) protein and mRNA levels were then detected via ELISA and qPCR, respectively. Moreover, the activation of pIGF-1R, pAKT, and pS6 was examined by Western blot analysis. To further explore the underlying mechanism, an IGF-1 neutralizing antibody, NVP-AEW541, and rapamycin were used. IGF-1, col-I, and col-III were significantly increased in stressed LFCs compared to nonstressed LFCs. In addition, the activation of pIGF-1R, pAKT, and pS6 was obviously enhanced in stressed LFCs. Interestingly, col-I protein, col-I mRNA, col-III protein, col-III mRNA, and IGF-1 protein, but not IGF-1 mRNA, were inhibited by IGF-1 neutralizing antibody. In addition, col-I and col-III protein and mRNA, but not IGF-1, were inhibited by both NVP-AEW541 and rapamycin. Moreover, the activation of pIGF-1R, pAKT, and pS6 was reduced by the IGF-1 neutralizing antibody and NVP-AEW541, and the activation of pS6 was reduced by rapamycin. In summary, these results suggested that mechanical stress promotes LFCs to produce IGF-1, which facilitates col-I and col-III synthesis via the IGF-1R/AKT/mTORC1 signaling pathway.

## 1. Introduction

Currently, an increasing number of elderly individuals have lumbar spinal stenosis (LSS) [1, 2]. The clinical symptoms of LSS include lower limb numbness with pain, low back pain, and claudication [3]. LSS causes tremendous discomfort for patients, and LSS is often caused by ligamentum flavum hypertrophy (LFH) [4, 5]. Previous studies [6–9] have shown that mechanical stress promotes collagen I (col-I) and collagen III (col-III) synthesis which contributes to LFH. However, the exact mechanisms remain unclear.

According to a previous study [10], IGF-1 is important for anabolism and stimulates the IGF-1R/AKT/mTORC1 signaling pathway, resulting in muscle or bone formation [11–13]. Moreover, increased IGF-1 promotes hypertrophy of various tissues [14–17], and mechanical stress plays a vital role in IGF-1 formation [18, 19]. We have previously reported that [20] exogenous IGF-1 promotes col-I and col-III synthesis in LFCs, which are fibrous connective tissue stem cells. However, in LFH, the relationship of mechanical stress and IGF-1 has not been sufficiently studied.

In the present study, we hypothesized that mechanical stress plays a pivotal role in IGF-1 synthesis. In addition, IGF-1 may promote col-I and col-III synthesis by the IGF-1R/AKT/mTORC1 signaling pathway. To test these hypotheses, col-I and col-III as the important indicator of LFH were detected, and related marker activation of the IGF-1R/AKT/mTORC1 signaling pathway was examined. In addition, the relationship of mechanical stress and IGF-1 in LFH as well as the potential mechanism involved was investigated.

## 2. Materials and Methods

### 2.1. LFC Cultivation and Identification

First, nonthickened ligamentum flavum (LF) samples were aseptically obtained from 9 lumbar surgery patients (5 males and 4 females with an average age of 47.2 years). The LF samples were washed with physiological saline 3 times before being minced into 0.5 mm^3^ pieces. The LF samples were then digested with 0.2% collagenase-I for 1.5 h at 37°C and washed 2 times with PBS. The LF samples were centrifuged 3 times at 1000 r/min for 5 min before being placed in cell culture plates with DMEM containing 20% fetal bovine serum (FBS). Subsequently, The LF samples were incubated, and the medium was changed every 3 days. Approximately one week later, LFCs migrated out from the LF samples. When the LFCs reached 80% confluence, they were passaged 1 : 2. Moreover, some of the LFCs were cryopreserved in media (10%DMSO + 20%FBS + 70%DMEM) at -80°C. LFC morphology was inspected, and the expression of vimentin and col-I in LFCs was detected by immunostaining [21].

### 2.2. Mechanical Stress Application

Experiments were performed with LFCs from each individual patient. The LFCs were grouped into experimental and control groups, and they were cultured in BioFlex I 6-well plates at 1 × 10^5^ cells per well. After LFCs reached 80% confluence, they were subjected to serum starvation (DMEM with 0.2% FBS) for 12 h. The experimental groups were subjected to cycles of relaxation for 10 s and 20% elongation for 10 s by a tension system (FX5K, Flexcell International Corporation, USA) [22–25] for 6 h, 12 h, and 24 h. Control groups were cultured in the same environment without mechanical stress.

### 2.3. IGF-1 Neutralizing Antibody Treatment

LFCs were grouped into the following 3 groups: nonstress group, stress group, and stress+IGF-1 neutralizing antibody (10 *μ*g/ml, Abcam, Cambridge, UK) group. LFCs in the stress group and the stress+IGF-1 neutralizing antibody group were subjected to cycles of relaxation for 10 s and 20% elongation for 10 s by the tension system for 24 h, and LFCs in the nonstress group were not subjected to mechanical stress. IGF-1, col-I and col-III protein, and mRNA levels were detected by ELISA and RT-qPCR, respectively, in each group. In addition, the activation of pIGF-1R, pAKT, and pS6 in each group was evaluated by Western blot analysis.

### 2.4. NVP-AEW541 Treatment

LFCs were grouped into the following 3 groups: nonstress group, stress group, and stress+100 ng/ml NVP-AEW541 (a specific inhibitor of IGF-1R, dissolved in DMSO, MedChem Express, Monmouth Junction, NJ) group. LFCs in the stress group and the stress+100 ng/ml NVP-AEW541 group were subjected to cycles of relaxation for 10 s and 20% elongation for 10 s by the tension system for 24 h, and LFCs in the nonstress group were not subjected to mechanical stress. IGF-1, col-I and col-III protein, and mRNA levels were detected by ELISA and RT-qPCR, respectively, in each group. Moreover, the activation of pIGF-1R, pAKT, and pS6 in each group was evaluated by Western blot analysis.

### 2.5. Rapamycin Treatment

LFCs were grouped into the following 3 groups: nonstress group, stress group, and stress+10 ng/ml rapamycin (a specific inhibitor of mTORC1, dissolved in DMSO, Alexis Biochemicals, Lausen, Switzerland) group. LFCs in the stress group and the stress+10 ng/ml rapamycin group were subjected to cycles of relaxation for 10 s and 20% elongation for 10 s by the tension system for 24 h, and LFCs in the nonstress group were not subjected to mechanical stress. IGF-1, col-I and col-III protein, and mRNA levels were detected by ELISA and RT-qPCR, respectively, in each group. Moreover, the activation of pS6 in each group was evaluated by Western blot analysis.

### 2.6. RT-qPCR Analysis

IGF-1, col-I, and col-III mRNA was measured by RT-qPCR in each group. First, we extracted total RNA from LFCs and detected its concentration and purity. Reverse transcription was then performed followed by qPCR. The primer sequences used in the present study are listed in Table 1 [21]. Sangon Biotech (Sangon Biotech, China) synthesized all primers in the study. All assays in the study were performed in triplicate. The samples were normalized to GAPDH and analyzed by the 2^-*ΔΔ*Cq^ method [26].

### 2.7. Enzyme-Linked Immunosorbent Assay

Culture supernatants from LFCs in each group were collected. To remove insoluble impurities and cell debris, the supernatants were centrifuged at 1000 g at 4°C for 20 min. The cleared supernatants were immediately used to measure IGF-1, col-I, and col-III protein levels by a Human IGF-1 ELISA Kit (Elabscience Biotechnology, China), Collagen I ELISA Kit (Elabscience Biotechnology, China), and Collagen III ELISA Kit (Elabscience Biotechnology), respectively.

### 2.8. Western Blot Assays

LFCs from each group were lysed on ice in lysis buffer, and the lysates were then added to Laemmli buffer at 100°C for 10 min. The LFC lysates were separated by electrophoresis, and the proteins were then transferred to nitrocellulose membranes. The membranes were blocked with TBS containing 5% nonfat milk for 2 h at 25°C. Subsequently, the membranes were incubated with primary antibodies for 12 h at 4°C followed by incubation with secondary antibodies for 1.5 h at 25°C. Finally, a chemiluminescence kit (Beyotime, China) was used to visualize the nitrocellulose membranes.

### 2.9. Statistical Analyses

Data were statistically analyzed and graphed using GraphPad Prism 5.01 (GraphPad Software Inc., San Diego, CA, USA). Protein and mRNA changes with or without mechanical stress at different times were analyzed by one-way ANOVA, and Tukey' s honestly significant difference was used as the post hoc method. The remaining data were analyzed by Student's *t*-test. The results were considered significant when *P* < 0.05, and the data are presented as the mean ± SD.

## 3. Results

### 3.1. Identification and Morphology of LFCs with or without Mechanical Stress

**Figure 1:**
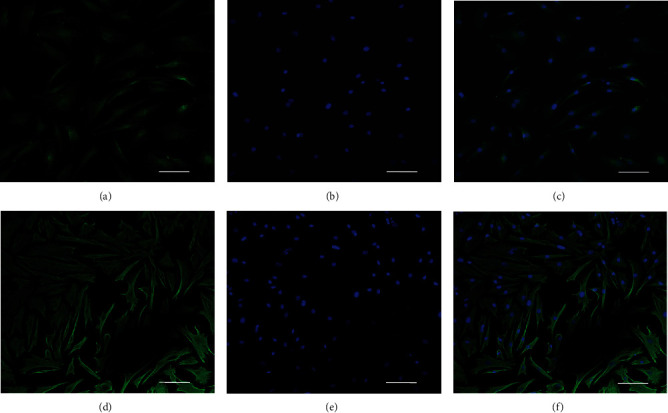
Identification of LFCs. Collagen I (a) and vimentin (d) immunofluorescence staining. Immunofluorescence is shown in green (a, d), and DAPI-stained nuclei are shown in blue (b, e). Merged pictures are shown (c, f). LFCs: ligamentum flavum cells; DAPI: 4′,6-diamidino-2-phenylindole. Scale bar = 50 *μ*m.

**Figure 2:**
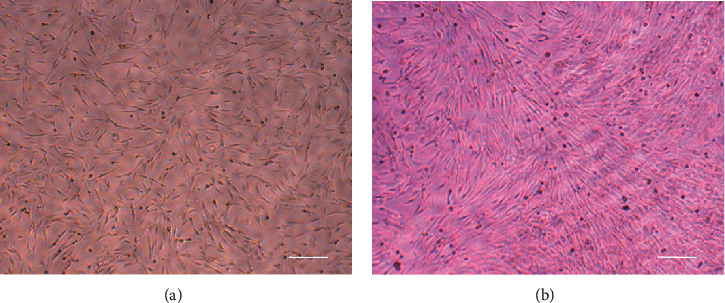
Morphology of LFCs with or without mechanical stress. LFCs were cultured without mechanical stress (a). LFCs were subjected to cycles of relaxation for 10 s and 20% elongation for 10 s (b). LFCs: ligamentum flavum cells. Scale bar = 100 *μ*m.

Immunofluorescence staining showed that LFCs expressed high levels of col-I and vimentin (Figure 1), which indicated that highly purified LFCs were cultured. Without mechanical stress, most LFCs were polygonal (Figure 2(a)). Under mechanical stress, LFCs became fusiform and arranged along the direction of stress (Figure 2(b)).

### 3.2. Mechanical Stress Promotes IGF-1, col-I, and col-III Protein and mRNA Production as well as Activation of pIGF-1R, pAKT, and pS6

**Figure 3:**
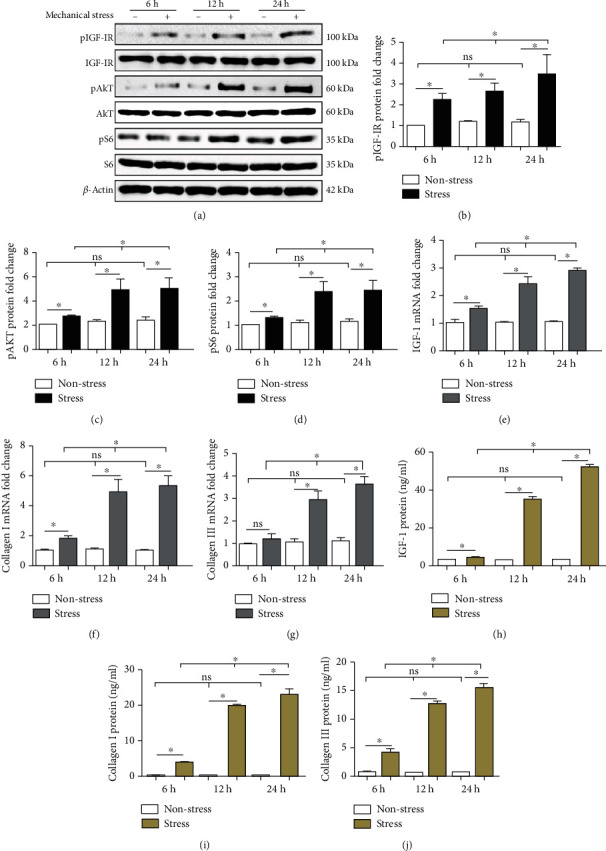
IGF-1R/AKT/mTORC1 signaling pathway-related mRNA and protein changes in LFCs with or without mechanical stress. Mechanical stress upregulated the activation of pIGF-1R (a, b), pAKT (a, c), and pS6 (a, d), as well as the mRNA levels of IGF-1 (e), col-I (f), and col-III (g) and the protein levels of IGF-1 (h), col-I (i), and col-III (j) in a time-dependent manner. Columns represent the mean ± SD of 3 samples, and each experiment was performed in triplicate. LFCs: ligamentum flavum cells; IGF-1: insulin-like growth factor 1; col-I: collagen I; col-III: collagen III. “∗” represents *P* < 0.05; “ns” indicates *P* > 0.05.

IGF-1, col-I, and col-III mRNA was examined via RT-qPCR in the stress group and the nonstress group at 6 h, 12 h, and 24 h. Mechanical stress upregulated IGF-1, col-I, and col-III mRNA production in a time-dependent manner (Figures 3(e)–3(g)). Moreover, IGF-1, col-I, and col-III protein levels were examined by ELISA at 6 h, 12 h, and 24 h. Mechanical stress increased IGF-1, col-I, and col-III protein production in a time-dependent manner (Figures 3(h)–3(j)). The activation of pIGF-1R, pAKT, and pS6 was evaluated in the stress group and the nonstress group by Western blot analysis at 6 h, 12 h, and 24 h. Mechanical stress increased the activation of pIGF-1R (Figures 3(a) and 3(b)), pAKT (Figures 3(a) and 3(c)), and pS6 (Figures 3(a) and 3(d)) in a time-dependent manner.

### 3.3. IGF-1 Neutralizing Antibody Reduces col-I and col-III mRNA Production; Reduces IGF-1, col-I, and col-III Protein Production; and Suppresses the Activation of pIGF-1R, pAKT, and pS6

**Figure 4:**
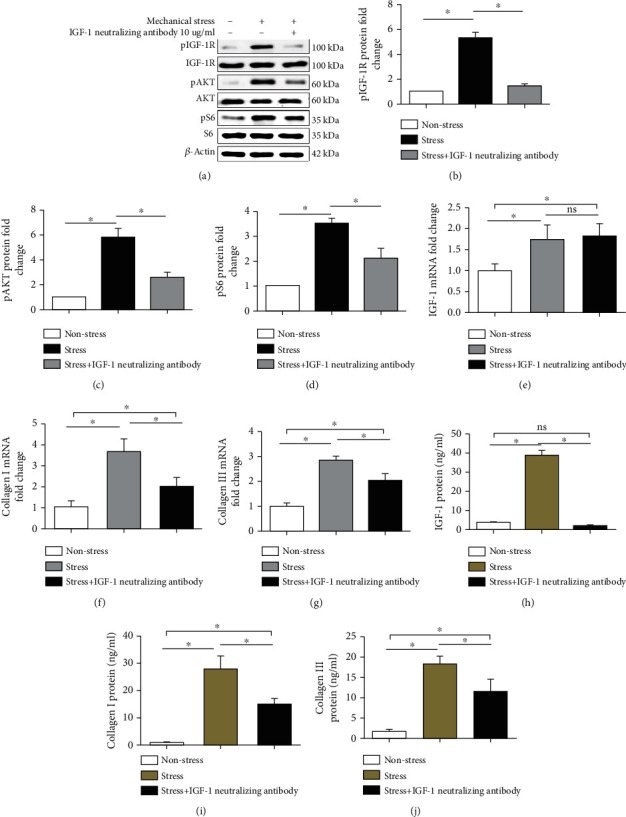
IGF-1 neutralizing antibody treatment. Mechanical stress in the nonstress group, the stress group, and the stress+10 *μ*g/ml IGF-1 neutralizing antibody group. The IGF-1 neutralizing antibody reduced the mRNA levels of col-I (f) and col-III (g), but not IGF-1 (e). The IGF-1 neutralizing antibody also reduced the protein expression of IGF-1 (h), col-I (i), and col-III (j). In addition, the activation of pIGF-1R (a, b), pAKT (a, c), and pS6 (a, d) was reduced by the IGF-1 neutralizing antibody. Columns represent the mean ± SD of 3 samples, and each experiment was performed in triplicate. LFCs: ligamentum flavum cells; IGF-1: insulin-like growth factor 1; col-I: collagen I; col-III: collagen III. “∗” represents *P* < 0.05; “ns” indicates *P* > 0.05.

IGF-1, col-I, and col-III mRNA and protein levels were examined by RT-qPCR and ELISA, respectively, at 24 h for the nonstress group, the stress group, and the stress+10 *μ*g/ml IGF-1 neutralizing antibody group. The IGF-1 neutralizing antibody reduced the mRNA levels of col-I/col-III (Figures 4(f) and 4(g)), but not IGF-1 (Figure 4(e)). Moreover, IGF-1, col-I, and col-III protein levels were reduced by the IGF-1 neutralizing antibody (Figures 4(h)–4(j)). Furthermore, IGF-1 neutralizing antibody suppressed the activation of pIGF-1R (Figures 4(a) and 4(b)), pAKT (Figures 4(a) and 4(c)), and pS6 (Figures 4(a) and 4(d)).

### 3.4. NVP-AEW541 Reduces IGF-1, col-I, and col-III Protein and mRNA Production and Suppresses the Activation of pIGF-1R, pAKT, and pS6

**Figure 5:**
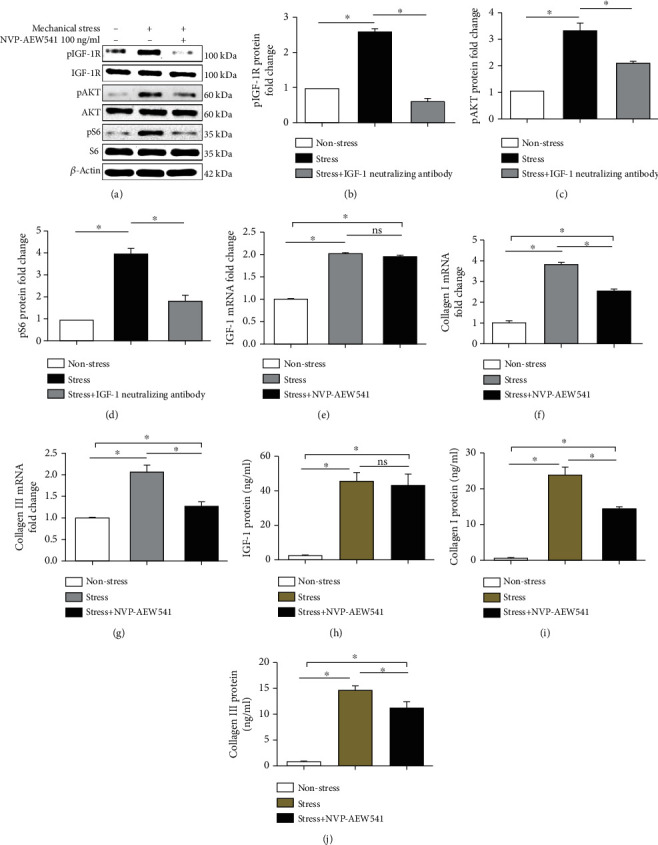
NVP-AEW541 treatment. Mechanical stress in the nonstress group, the stress group, and the stress+100 ng/ml NVP-AEW541 group. NVP-AEW541 reduced the mRNA levels of col-I (f) and col-III (g), but not IGF-1 (e). col-I (i) and col-III (j) protein levels, but not IGF-1 (h) protein levels, were attenuated by NVP-AEW541. In addition, NVP-AEW541 reduced the activation of pIGF-1R (a, b), pAKT (a, c), and pS6 (a, d). Columns represent the mean ± SD of 3 samples, and each experiment was performed in triplicate. LFCs: ligamentum flavum cells; IGF-1: insulin-like growth factor 1; col-I: collagen I; col-III: collagen III. “∗” represents *P* < 0.05; “ns” indicates *P* > 0.05.

IGF-1, col-I, and col-III mRNA and protein levels were examined by RT-qPCR and ELISA, respectively, at 24 h in the nonstress group, the stress group, and the stress+100 ng/ml NVP-AEW541 group. NVP-AEW541 reduced the mRNA levels of col-I/col-III mRNA (Figures 5(f) and 5(g)), but not IGF-1 (Figure 5(e)), and it reduced the protein levels of col-I/col-III protein (Figures 5(i) and 5(j)), but not IGF-1 (Figure 5(h)). In addition, the activation of pIGF-1R (Figures 5(a) and 5(b)), pAKT (Figures 5(a) and 5(c)), and pS6 (Figures 5(a) and 5(d)) was reduced by NVP-AEW541.

### 3.5. Rapamycin Reduces IGF-1, col-I, and col-III Protein and mRNA Production and Suppresses the Activation of pS6

**Figure 6:**
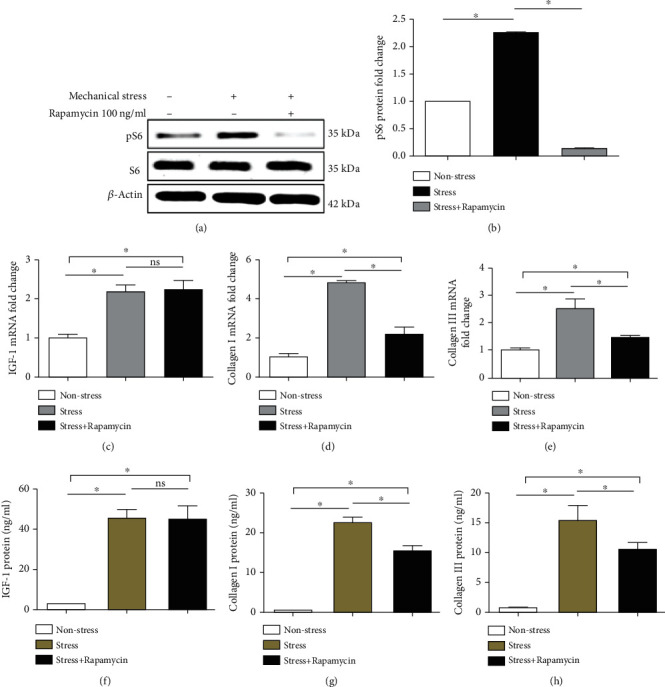
Rapamycin treatment. Mechanical stress in the nonstress group, the stress group, and the stress+rapamycin group. Rapamycin reduced the mRNA levels of col-I (d) and col-III (e), but not IGF-1 (c). Rapamycin reduced the protein levels of col-I (g) and col-III (h), but not IGF-1 (f). In addition, rapamycin reduced the activation of pS6 (a, b). Columns represent the mean ± SD of 3 samples, and each experiment was performed in triplicate. LFCs: ligamentum flavum cells; IGF-1: insulin-like growth factor 1; col-I: collagen I; col-III: collagen III. “∗” represents *P* < 0.05; “ns” indicates *P* > 0.05.

IGF-1, col-I, and col-III mRNA and protein levels were detected by RT-qPCR and ELISA, respectively, at 24 h in the nonstress group, the stress group, and the stress+10 ng/ml rapamycin group. Rapamycin decreased the mRNA levels of col-I/col-III (Figures 6(d) and 6(e)), but not IGF-1 (Figure 6(c)), and it reduced the protein levels of col-I/col-III (Figures 6(g) and 6(h)), but not IGF-1 (Figure 6(f)). In addition, the induction of pS6 was suppressed by rapamycin (Figures 6(a) and 6(b)).

## 4. Discussion

In previous studies, LFH has been identified as a common cause of LSS [3–5]. LFH is caused by increased collagen levels, mainly col-I and col-III [8, 21, 27–29]. Many inflammatory and growth factors such as IL-1, IL-6, TGF-*β*1, VEGF, PDGF-BB, CTGF, and TNF-*α* have been reported to promote col-I and col-III production, eventually leading to LFH [8, 21, 27, 30–36]. Chuang et al. [37] showed that oxidative stress activates the Akt and MAPK pathways to upregulate inflammatory mediator (iNOS and NF-*κ*B) and fibrotic marker (TGF-*β*, *β*-catenin, *α*-SMA, and vimentin) expression levels, thereby contributing to LFH. Habibi et al. [38] confirmed that acidic fibroblast growth factor (FGF-1) expression is higher in LSS patient tissues than in nonhypertrophied ligamentum flavum tissues.

IGF-1 is a vital growth factor that promotes collagen production via the mTORC1 signaling pathway [11–13]. According to previous studies [14–17], IGF-1 is released by various types of cells under mechanical stress and IGF-1 increases collagen expression levels, which contributes to the hypertrophy of various tissues. Some studies have also reported [6–9] that mechanical stress may play a vital role in LFH. Nakatani et al. [8] indicated that mechanical stress stimulates LFCs to produce TGF-*β*l, which increases the synthesis of collagens, resulting in LFH. Hayashi et al. [9] reported that fibroblast growth factor 9 (FGF9) and its pathway contribute to LFH under mechanical stress. Reijnders et al. [18] reported that mechanical stress results in IGF-1 mRNA upregulation in osteocytes of rat tibia and that IGF-1 is involved in the translation of mechanical stress to bone formation. Juffer et al. [19] showed that mechanical stress stimulates MLO-Y4 osteocytes to express IGF-1 isoform, which is an important factor in anabolism and metabolism in muscle, at the mRNA and protein levels. However, the interaction between mechanical stress and IGF-1 has not been previously studied in LFH. In the present study, we researched the correlation of mechanical stress, IGF-1, and the IGF-1R/AKT/mTORC1 signaling pathway in LFH.

First, we isolated primary LFCs from 9 patients who underwent lumbar spinal surgery. LFCs are fibrous connective tissue stem cells. According to the study conducted by Zhong and Chen [39], LFCs can be identified by detecting col-I and vimentin expression. Therefore, in the present study, LFC purity was examined by col-I and vimentin expression. Immunofluorescence results showed that the LFCs were of high purity (Figure 1). In addition, LFC viability was evaluated by the MTT assay (Solarbio, China), which demonstrated that there were no changes in LFC viability in each group.

LFCs were also subjected to cyclic mechanical stress at different times. Compared to nonstressed cells, cyclic mechanical stress promoted the synthesis of IGF-1 in LFCs in a time-dependent manner, which eventually led to col-I/col-III accumulation via the IGF-1R/AKT/mTORC1 signaling pathway. To understand the molecular mechanism involved, we used an IGF-1 neutralizing antibody. Compared to the nonstress group, IGF-1, col-I, and col-III protein and mRNA levels were increased in the stress group. In addition, col-I and col-III protein and mRNA levels were significantly reduced in the stress+IGF-1 neutralizing antibody group compared to the stress group. Interestingly, IGF-1 protein expression, but not mRNA expression, was reduced in the stress+IGF-1 neutralizing antibody group compared to the stress group. Correspondingly, the activation of pIGF-1R, pAKT, and pS6 was decreased in the stress+IGF-1 neutralizing antibody group compared to the stress group. For further investigation, NVP-AEW541 (a specific inhibitor of IGF-1R) and rapamycin (a specific inhibitor of mTORC1) were used in the present study. Although 100 ng/ml NVP-AEW541 and 10 ng/ml rapamycin almost completely blocked the IGF-1R/AKT/mTORC1 signaling pathway, col-I and col-III protein and mRNA levels were only partially reduced. col-I and col-III protein and mRNA levels were still higher in both groups compared to the nonstress group. Moreover, neither NVP-AEW541 nor rapamycin reversed IGF-1 expression, which was induced by mechanical stress.

Based on the above findings, we hypothesized that mechanical stress may promote col-I and col-III production via other signaling pathways, and the potential mechanism involved requires further study. In addition, due to the lack of an animal model of LFH, only cytological experiments were performed in the present study. Thus, it is necessary to build an effective animal model for further research.

## 5. Conclusion

**Figure 7:**
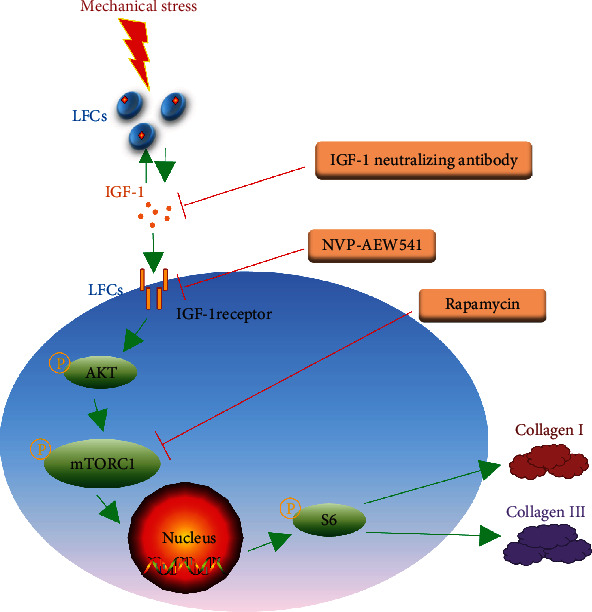
Potential mechanism by which mechanical stress promotes collagen I and collagen III synthesis.

In summary, the present study showed that mechanical stress upregulated IGF-1, col-I, and col-III protein and mRNA production. The IGF-1 neutralizing antibody, NVP-AEW541, and rapamycin blocked the IGF-1R/AKT/mTORC1 signaling pathway and reduced col-I and col-III production in LFCs. These findings demonstrated that cyclic mechanical stress promotes LFCs to secrete IGF-1, which induces col-I and col-III synthesis via the IGF-1R/AKT/mTORC1 signaling pathway (Figure 7). These results provide a new understanding of LFH and may facilitate the development of novel methods to treat LSS.

## Figures and Tables

**Table 1:** Primers used in the study.

Gene	Sequence (5′ to 3′)
IGF-1	Forward GTG TTG CTT CCG GAG CTG TG
	Reverse CAA ATG TAC TTC CTT CTG AGT C
Collagen I	Forward GTC GAG GGC CAA GAC GAA G
	Reverse CAG ATC ACG TCA TCG CAC AAC
Collagen III	Forward ATG TTC CAC GGA AAC ACT GG
	Reverse GGA GAG AAG TCG AAG GAA TGC
GAPDH	Forward ACA CCC ACT CCT CCA CCT TT
	Reverse TTA CTC CTT GGA GGC CAT GT

## Data Availability

Data are available on request (detail contact information: nfzzm@163.com).
